# The Value of Vaccination in Older Adults: The Why

**DOI:** 10.1093/geroni/igaa057.2929

**Published:** 2020-12-16

**Authors:** Philip Buck

**Affiliations:** GSK Vaccines, Philadelphia, Pennsylvania, United States

## Abstract

The incidence of vaccine-preventable diseases remains high among older adults in the US, despite longstanding immunization recommendations, and is projected to increase as the population ages. The impact of US population aging on the burden of four vaccine-preventable diseases (influenza, pneumococcal disease, shingles, and pertussis) was modeled over a 30-year time horizon, with cumulative direct and indirect costs increasing from $378 billion over 10 years to $1.28 trillion over 30 years. Compared to current levels of vaccination coverage, increasing coverage was predicted to avert over 33 million cases of disease and greater than $96 billion in disease-associated costs, with a corresponding increase in vaccination costs of approximately $83 billion over the entire 30-year time period. Specific examples of cost-effectiveness analyses that assess the epidemiologic and economic impact of vaccination against shingles and pertussis in older adults will be discussed. Part of a symposium sponsored by the Health Behavior Change Interest Group.

